# Estimation of disease burden and clinical severity of COVID-19 caused by Omicron BA.2 in Shanghai, February-June 2022

**DOI:** 10.1080/22221751.2022.2128435

**Published:** 2022-11-21

**Authors:** Xinhua Chen, Xuemei Yan, Kaiyuan Sun, Nan Zheng, Ruijia Sun, Jiaxin Zhou, Xiaowei Deng, Tingyu Zhuang, Jun Cai, Juanjuan Zhang, Marco Ajelli, Hongjie Yu

**Affiliations:** a Shanghai Institute of Infectious Disease and Biosecurity, School of Public Health, Fudan University, Shanghai, People’s Republic of China; b Key Laboratory of Public Health Safety, Ministry of Education, Fudan University, Shanghai, People’s Republic of China; c Division of International Epidemiology and Population Studies, Fogarty International Center, National Institutes of Health, Bethesda, MD, USA; d Laboratory for Computational Epidemiology and Public Health, Department of Epidemiology and Biostatistics, Indiana University School of Public Health, Bloomington, IN, USA

**Keywords:** Omicron BA.2, COVID-19, disease burden, clinical severity, Shanghai

## Abstract

An outbreak of COVID-19 caused by the SARS-CoV-2 Omicron BA.2 sublineage occurred in Shanghai, China from February 26 to June 30, 2022. We use official reported data retrieved from Shanghai municipal Health Commissions to estimate the incidence of infections, severe/critical infections, and deaths to assess the disease burden. By adjusting for right censoring and RT-PCR sensitivity, we provide estimates of clinical severity, including the infection fatality ratio, symptomatic case fatality ratio, and risk of developing severe/critical disease upon infection. The overall infection rate, severe/critical infection rate, and mortality rate were 2.74 (95% CI: 2.73-2.74) per 100 individuals, 6.34 (95% CI: 6.02-6.66) per 100,000 individuals and 2.42 (95% CI: 2.23-2.62) per 100,000 individuals, respectively. The severe/critical infection rate and mortality rate increased with age, noted in individuals aged 80 years or older. The overall fatality ratio and risk of developing severe/critical disease upon infection were 0.09% (95% CI: 0.09-0.10%) and 0.27% (95% CI: 0.24-0.29%), respectively. Having received at least one vaccine dose led to a 10-fold reduction in the risk of death for infected individuals aged 80 years or older. Under the repeated population-based screenings and strict intervention policies implemented in Shanghai, our results found a lower disease burden and mortality of the outbreak compared to other settings and countries, showing the impact of the successful outbreak containment in Shanghai. The estimated low clinical severity of this Omicron BA.2 epidemic in Shanghai highlight the key contribution of vaccination and availability of hospital beds to reduce the risk of death.

## Introduction

An outbreak of coronavirus disease 2019 (COVID-19) caused by the severe acute respiratory syndrome coronavirus 2 (SARS-CoV-2) Omicron BA.2 sublineage has occurred in Shanghai, China from February to June 2022. To contain the outbreak in the shortest period of time, the Shanghai government has implemented a set of strict nonpharmacological interventions (NPIs). Between March 16 and March 27, Shanghai adopted grid management at the subdistrict level, fractioning subdistricts into high-risk areas and nonhigh-risk areas according to the number of cases and infections in the area. In addition, several rounds of population-wide nucleic acid test screenings were performed within high-risk areas and nonhigh-risk areas. However, such interventions were not sufficient to control community spread. The entire city was forced to enter a phased stage of lockdowns starting on March 28 when Shanghai's Pudong district entered a population-wide lockdown, and the entire city entered a lockdown phase on April 1, 2022. The epidemic reached its inflection point on April 13, 2022, and the outbreak was eventually brought under control. On June 1, 2022, the Shanghai government declared the end of the city-wide lockdown. At that time, the total number of reported infections was 626,811, including 568,811 asymptomatic infections, 58,000 symptomatic cases, and 588 deaths. [1]

One of the reasons that prompted Shanghai to adopt stringent NPIs was the vaccination gap in Shanghai's elderly population. In fact, as of April 15, 2022, only 62% and 38% of people older than 60 years old completed primary and booster vaccinations, respectively. [2] The generally low coverage of primary and booster vaccination among the elderly – the population at highest risk of severe and critical COVID-19 disease – could have led to a large number of deaths if the outbreak were left to spread uncontrolled. In comparison, an uncontained Omicron outbreak in Hong Kong, China resulted in 9,188 deaths between December 31, 2021 and June 30, 2022. [3] In addition to the relatively low vaccination coverage in the elderly, the inactivated vaccines widely used in Shanghai were found to have a low effectiveness. Specifically, in a study conducted in Hong Kong during the Omicron BA.2 wave, the estimated primary and booster vaccine effectiveness against mild or moderate Omicron BA.2 infections were 25.1% (95% CI:14.7-34.3%) and 51.0% (95% CI: 39.6-60.4%) for CoronaVac inactivated vaccine, respectively. [4] Moreover, the same study found that inactivated vaccines were inferior across all clinical severity end points as compared to mRNA vaccines.

Estimation of disease burden and clinical severity are critical to identify appropriate intervention strategies, plan for healthcare needs, and ensure that the health system operates properly. Recent studies have investigated specific metrics of Omicron severity; specifically, in-hospital mortality,[5] risk of hospital admission, intensive care unit admission, mechanical ventilation, and death,[6] and case fatality risk. [7] However, a comprehensive characterization of the disease burden and clinical severity of Omicron BA.2 variant is still lacking, especially in populations predominately immunized with inactivated vaccines. During the 2022 outbreak in Shanghai, the government organized multiple rounds of molecular test screenings for the entire population, which led to a high infection ascertainment ratio, thus providing a unique opportunity to capture the majority of subclinical infections and better characterize the full spectrum of Omicron BA.2 clinical severity.

In this study, we used publicly reported data from the Shanghai Municipal Health Commission to estimate the incidence of infections, severe/critical infections, and deaths to assess the disease burden of the Omicron BA.2 outbreak in Shanghai between February and June 2022. In addition, we provide estimates of the infection fatality ratio and symptomatic case fatality ratio as well as the risk of developing severe/critical disease upon infection to assess the clinical severity of Omicron BA.2 during the outbreak.

## Materials and methods

### Case definition

The definition of confirmed COVID-19 cases and SARS-CoV-2 infection was based on the Clinical Guidance for COVID-19 Pneumonia Diagnosis and Treatment (trial ninth edition) and the COVID-19 Prevention and Control Protocol (eighth edition) published by the National Health Commission (NHC) of China. [8, 9] SARS-CoV-2 infections, including asymptomatic infections and symptomatic cases, were ascertained by Reverse Transcription-Polymerase Chain Reaction (RT–PCR). Two types of RT–PCR kits were used for case detection and massive nucleic acid screening during the outbreak (BioGerm, Lot No. 20200304A; Bioperfectus, Lot No. JC10223-1N). Symptomatic cases were further categorized by clinical severity into mild, moderate, severe, and critical cases. Mild cases were defined as having mild symptoms, such as fever, fatigue, loss of taste/smell but without radiographic evidence of pneumonia. Moderate cases were those with typical symptoms of a respiratory infection (e.g. fever, dry cough, fatigue, etc.) and radiographic evidence of pneumonia. Severe cases referred to those patients with at least one of the following conditions: breathing problems, low oxygen saturation, low PaO_2_/FiO_2_ (PaO_2_ denotes partial pressure of oxygen in arterial blood; FiO_2_ denotes fraction of inspired oxygen), or progressive symptoms combined with pulmonary imaging showing obvious progress of lesions (>50%) within 24-48 h. Critical cases referred to patients who met any one of the following three criteria: respiratory failure, shock, or organ failure that required intensive care unit admission. [8] Asymptomatic infections were defined as RT–PCR-positive individuals who did not meet any of the following clinical criteria: 1) fever, cough, sore throat, and other self-perceived and clinically identifiable symptoms or signs; 2) no radiographic evidence of pneumonia. Cases who received at least one dose of vaccine were defined as the status of “vaccinated”. Cases who have no history of COVID-19 vaccination were defined as “unvaccinated”.

### Case identification and surveillance

During the Omicron BA.2 outbreak in Shanghai, multiple mass population screenings using RT–PCR tests were conducted for the entire city population to identify infected individuals, including asymptomatic and presymptomatic individuals (i.e. individuals who were asymptomatic at the time of diagnosis but subsequently developed symptoms) **(Figure S1)**. [10, 11] Moreover, self-performed rapid antigen test screenings of the population were performed as a supplement of nucleic acid tests; any positive result of an antigen test required confirmation by a nucleic acid test. Routine surveillance was mainly based on symptom-based surveillance from medical institutions. Contact tracing, epidemiological investigation, and screening of high-risk populations were also conducted.

### Data sources and parameters

Daily reports on the cumulative number of infections, symptomatic cases, and deaths were extracted from the websites of the Shanghai Municipal Health Commission since February 26, 2022, which represents the beginning of the Omicron BA.2 outbreak in Shanghai. [1] The distribution of age and vaccination status of infections, severe/critical patients, and deaths before May 13, 2022 were extracted from the literature. [12] The data of deceased cases after May 13, 2022 were extracted from the report of Shanghai Municipal Health Commissions. [1] All data sources and parameters used are listed in **Table S1**.

### Primary outcomes

**Figure 1.**
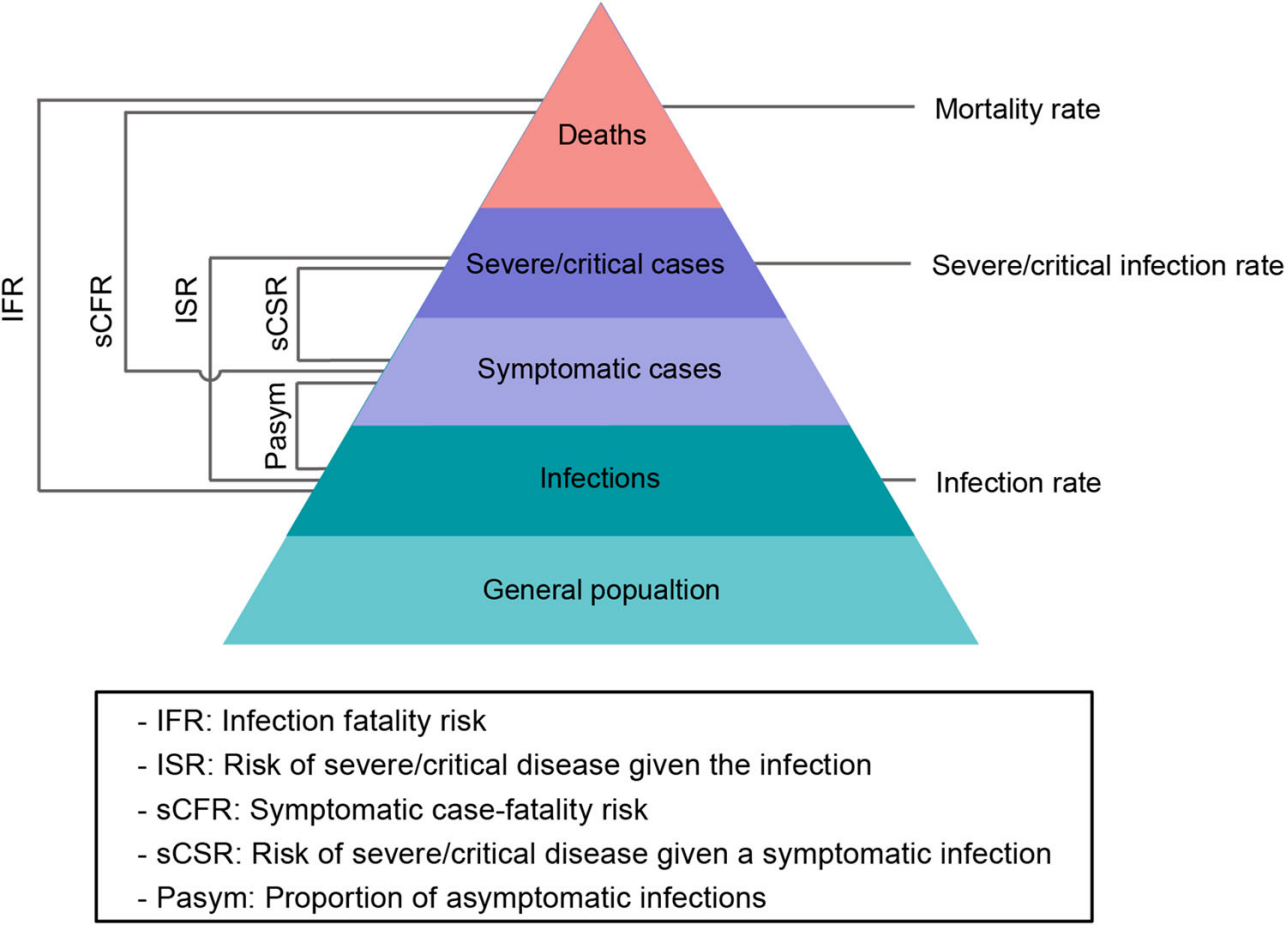
Spectrum of COVID-19 and primary outcomes.

To assess the burden of the investigated outbreak, we estimated the prevalence of infections, cases of severe/critical disease, and deaths. To assess the clinical severity of the Omicron BA.2 variant during the investigated outbreak, we estimated the proportion of asymptomatic infections (P_asym_), infection fatality ratio (IFR), risk of severe/critical disease given the infection (ISR), symptomatic case-fatality ratio (sCFR), and risk of severe/critical disease given a symptomatic infection (sCSR). Specifically, the P_asym_ was defined as the ratio between the number of asymptomatic infections and the total number of infections. The IFR was defined as the ratio between the number of deaths and the total number of SARS-CoV-2 infections, which includes both asymptomatic infections and symptomatic cases confirmed by RT–PCR. ISR was defined as the ratio between the number of severe/critical cases and the total number of SARS-CoV-2 infections confirmed by RT–PCR. The sCFR was defined as the ratio between the number of deaths and the number of symptomatic COVID-19 cases confirmed by RT–PCR. The sCSR was defined as the ratio between the number of severe/critical cases and the number of symptomatic COVID-19 cases confirmed by RT–PCR (Figure 1). [13–15]

### Statistical analysis

#### Estimation of the disease burden

Given that the cumulative number of severe/critical cases at the end of the outbreak was not officially reported, we extracted the number of severe/critical cases before May 13 from the literature (*Wang et al., personal communication*). Based on the assumption that the risk of death among severe/critical patients remained constant from May 13 until the end of the outbreak, we used the age-specific risk to estimate the cumulative number of severe/critical infections given the cumulative number of deaths by age category. We estimated a set of age-specific attack rates between February 26 and June 30, 2022 by dividing the total number of reported infections, severe/critical patients, and deaths by the population in each age group. Fisher's exact test was used to test difference between age groups.

#### Estimation of clinical severity

We estimated both crude and adjusted P_asym_, IFR, ISR, sCFR, and sCSR to assess the clinical severity of Omicron BA.2 during the Shanghai outbreak. Using Garkse's method,[16] we adjusted for right censoring by weighting the denominator with the distribution of the time interval from infection to symptom onset, infection to severe/critical illness, infection to death, symptom onset to severe/critical illness, and symptom onset to death (**Equations 1-5, see** Figure 1 **for better representation**).

(1) $$P_{asym}(t_{2})=1-\;\frac{Sy(t_{2})}{\sum_{t_{1}=0}^{t_{2}}F(t_{2}-t_{1})C(t_{1})}$$

(2) $$IFR(t_{2})=\frac{D(t_{2})}{\sum_{t_{1}=0}^{t_{2}}F(t_{2}-t_{1})C(t_{1})}$$

(3) $$ISR(t_{2})=\frac{Se(t_{2})}{\sum_{t_{1}=0}^{t_{2}}F(t_{2}-t_{1})C(t_{1})}$$

(4) $$sCFR(t_{2})=\frac{D(t_{2})}{\sum_{t_{1}=0}^{t_{2}}F(t_{2}-t_{1})S(t_{1})}$$

(5) $$sCSR(t_{2})=\frac{Se(t_{2})}{\sum_{t_{1}=0}^{t_{2}}F(t_{2}-t_{1})S(t_{1})}$$

where the numerator refers to the cumulative number of cases with predefined endpoints on the cut-off date of the analysis, *t*_2_ (Sy: symptomatic infection; Se: severe and critical infection; D: death), and the denominator refers to a weighted sum of daily (*t*_1_) reported total number of confirmed infections **(Equations 1-3)** or symptomatic cases **(Equations 4-5)**. The weights are based on the density distribution of the time interval from infection to symptom onset, from infection to severe/critical illness, from infection to death, from symptom onset to severe/critical illness, and from symptom onset to death (F). Essentially, this design allows the exclusion of a proportion of recent cases for whom the final outcome has yet to be observed at the early stage of outbreak. To obtain the interval parameter from infection to death, we resampled three intervals (interval from infection to symptom onset; onset to admission; admission to death) according to studies that reported estimated distributions of key time to event of Omicron BA.2 (*Yu et al., unpublished data*). Then, we add these three intervals for each individual category and fit gamma, lognormal, and Weibull distributions to identify the best estimates according to the Akaike Information Criterion (AIC) and the Bayesian Information Criterion (BIC). We used a similar method to estimate the interval from infection to severe/critical illness. Additionally, we further adjusted the number of total infections by randomly simulating 10,000 draws of RT-PCR sensitivity from a binomial distribution to test the uncertainty of the parameter regulating the RT-PCR sensitivity. We further estimated the IFR and ISR stratified by age groups (3-17, 18-39, 40-59, 60-79, and ≥ 80 years) and vaccination status (not vaccinated and vaccinated). Binomial distributions were used to estimate 95% CIs.

#### Sensitivity analysis

As mentioned above, two types of RT-PCR kits were used for case detection, but their actual usage and distribution is unknown. In the main analysis, we used RT-PCR sensitivity from a single manufacturer (BioGerm). We then conducted a sensitivity analysis by accounting for the sensitivity of the second manufacturer (Bioperfectus) and re-estimated disease burden and clinical severity.

#### Correlation analysis

We conducted a rapid literature review and comprehensively collected studies reporting IFR and/or sCFR in other settings with predefined search terms **(Table S2)**. Then, we combined the retrieved literature with our estimations and used a linear regression model to explore the impact of vaccine coverage of primary and booster vaccinations to the IFR and sCFR. The Pearson correlation coefficient and the corresponding *p* value were reported for each correlation analysis.

## Results

### Disease burden of COVID-19 caused by Omicron BA.2

**Figure 2.**
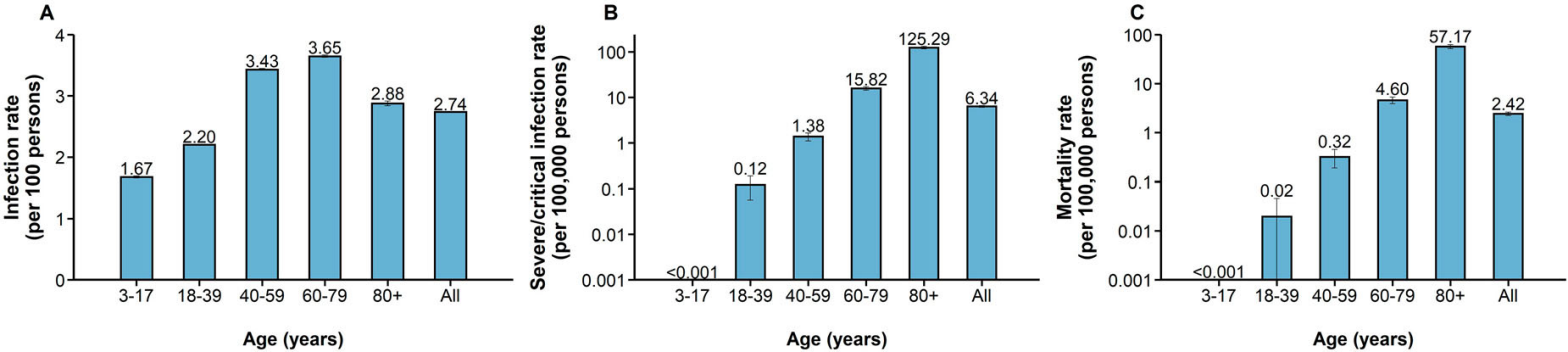
**Infection rate, severe/critical infection rate and mortality rate of COVID-19 caused by SARS-CoV-2 Omicron in Shanghai A)** Infection rate (per 100 individuals); **B)** Severe/critical infection rate (per 100,000 individuals); **C)** Mortality rate (per 100,000 individuals).

From February 26 to June 30, 2022, a total of 627,115 confirmed infections were officially reported, including 58,137 symptomatic cases, 568,978 asymptomatic infections, and 588 deceased cases. The overall infection rate was 2.74 (95% CI: 2.73-2.74) per 100 individuals. The lowest infection rate was noted in the population aged 3–17 years (1.67, 95% CI: 1.66-1.69 per 100 individuals), whereas the highest rate in the population aged 60–79 years (3.65, 95% CI: 3.63-3.67 per 100 individuals). (Figure 2A). The sensitivity analysis showed the same pattern (**Figure S2**). The rate of severe/critical infection increased with age. The highest rate of 125.29 (95% CI: 117.05-133.44) per 100,000 individuals was noted in individuals aged 80 years or older, which was significantly higher than <0.001 per 100,000 individuals aged 3–17 years (*p* < 0.0001) (Figure 2B). The overall mortality rate was 2.42 (95% CI: 2.23-2.62) per 100,000 individuals. Regarding specific age groups, the mortality rate was 0 in 3- to 17-year-old individuals, 0.02 (95% CI: 0-0.04) per 100,000 individuals in those aged 18–39 years, 0.32 (95% CI: 0.19-0.45) per 100,000 individuals in those aged 40–59 years, 4.60 (95% CI: 3.87-5.32) per 100,000 individuals in those aged 60–79 years, and 57.17 (95% CI: 51.63-62.71) per 100,000 individuals in those aged 80 years or older, with significantly higher mortality in those aged 80 years or older than other age groups (*p* < 0.0001) (Figure 2C).

### Clinical severity of COVID-19 caused by Omicron BA.2

**Figure 3.**
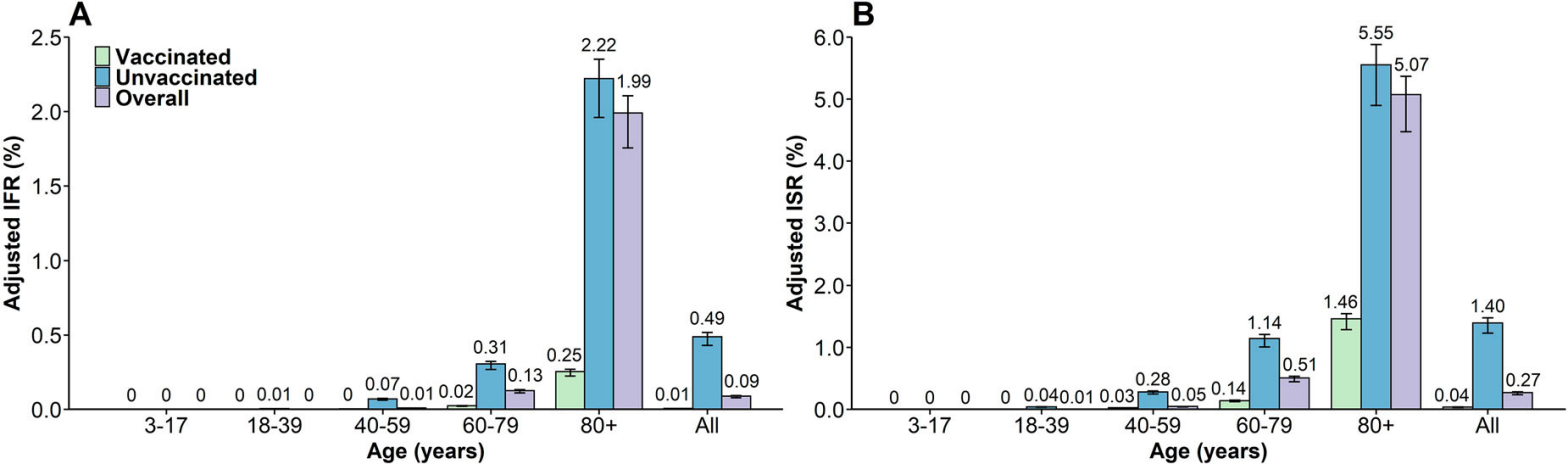
Adjusted IFR and ISR of COVID-19 caused by SARS-CoV-2 Omicron in Shanghai **A)** IFR; **B)** ISR. The number represents the median estimates, and the error bar represents the 95% confidence interval.

By June 30, 2022, the adjusted P_asym_ was 90.7% (95% CI: 90. 7-90.8%) **(Figure S3)**. The overall adjusted IFR was 0.09% (95% CI: 0.09-0.10%) with an extremely low risk of fatality for infected individuals younger than 60 years **(**Figure 3A**, Figure S4)**. The adjusted IFR was 0.13% (95% CI: 0.11-0.14%) for individuals aged 60–79 and 1.99% (95% CI: 1.76-2.11%) for those aged 80 years or older. The adjusted IFR for unvaccinated infected individuals aged 80 years or older (corresponding to 88.2% of the total infections aged 80 + years) was 2.22% (95% CI: 1.96-2.35%), which was approximately 10-fold higher than that of those who had received at least one COVID-19 vaccine dose, namely, 0.25% (95% CI: 0.20-0.24%). The adjusted IFR for individuals aged 60–79 years was 0.02% (95% CI: 0.02-0.03%) for vaccinated individuals and 0.31% (95% CI: 0.27-0.32%) for unvaccinated individuals (Figure 3A). The overall adjusted ISR was 0.27% (95% CI: 0.24-0.29%). The highest risk of 5.08% (95% CI: 4.48-5.38%) was estimated for individuals aged 80 years or older, whereas this value was approximately 0 in the youngest age groups (e.g. 3–17 years). For individuals aged 80 years or above, the adjusted ISR in the vaccinated group (1.46%, 95% CI: 1.29-1.55%) was significantly lower than that in the unvaccinated group (5.56%, 95% CI: 4.90-5.89%) (*p* < 0.0001) (Figure 3B). Sensitivity analysis showed the change of RT-PCR's sensitivity will not affect the severity estimates **(Figure S5)**. The adjusted sCFR and sCSR were 0.96% (95% CI: 0.84-1.01%) and 2.97% (95% CI: 2.62-3.14%), respectively.

### Correlation analysis between IFR and vaccine coverage

From the analysis of estimates available in the literature (including the estimates provided in the current study), we found that the IFR was lower in settings with higher vaccination coverage of either primary or booster doses regardless of the circulating SARS-CoV-2 lineage/sublineage **(Figure S6)**. Specifically, by limiting the analysis to the Omicron variant, we found a significant correlation between the IFR and the coverage of the primary series of COVID-19 vaccine (Pearson correlation coefficient: −0.99; *p* = 0.015) **(Figure S7)**. We further stratified such estimates by age groups by correlating age-specific coverages across different locations and found a similar pattern **(Figure S8)**.

## Discussion

We estimated the disease burden and clinical severity of the SARS-CoV-2 Omicron BA.2 outbreak in Shanghai that occurred between February 26 and June 30, 2022. We estimated that the infection rate, severe/critical infection rate, and mortality rate were 2.74 (95% CI: 2.73-2.74) per 100 individuals, 6.34 (95% CI: 6.02-6.66) per 100,000 individuals and 2.42 (95% CI: 2.23-2.62) per 100,000 individuals, respectively. The severe/critical infection rate and mortality rate were both significantly higher in the elderly population compared with the remainder of the population, and these values were approximately 0 among young individuals (18 years or below). The overall IFR and ISR were 0.09% (95% CI: 0.09-0.10%) and 0.27% (95% CI: 0.24-0.29%), respectively. Having received at least one vaccine dose led to a 10-fold reduction in the risk of death for infected individuals aged 80 years or above.

The Omicron BA.2 outbreak in Shanghai had an intensive community-level transmission, requiring the implementation of strict NPIs. The outbreak was brought under control through a city-level lockdown, traffic shutdown, school closure, repeated and extensive nucleic screenings and medical assistance from other provinces. [17] This policy minimized the disease burden and number of fatalities. In fact, compared to the disease burden of COVID-19 caused by the Omicron variant in other countries, the overall infection rate and mortality rate in Shanghai were much lower. For example, the infection rate in Shanghai was 2.74 per 100 individuals compared to 34.37 per 100 individuals in South Korea, 13.67 per 100 individuals in Spain, and 24.13 per 100 individuals in Germany. However, in these countries, the number of infected individuals was largely underestimated because population mass screenings were not adopted. A study in Gauteng, South Africa estimated the reported deaths and excess deaths associated with COVID-19 during the Omicron wave to be 7 and 12 per 100,000 individuals, respectively. [18] Despite the younger South African population (median age: 29 years[19]), these estimates are remarkably higher compared to the mortality rate of 2.42 per 100,000 we estimated for Shanghai (median age: 38[20]). Since January 2022, Hong Kong has also experienced a large-scale epidemic caused by the Omicron BA.2 variant. As of June 30, 2022, greater than 1,233,166 infections and 9,188 deaths have been reported by the Hong Kong Health Agency, corresponding to infection and mortality rates of 1,665.0 per 10,000 individuals and 124.1 per 100,000 individuals, respectively. [3, 21, 22] The same Omicron variant caused both the Shanghai and Hong Kong outbreaks, and the remarkable difference in mortality could likely be ascribable to the difference in the adopted control measures. In fact, in contrast with Shanghai, the interventions adopted in Hong Kong were less strict and did not aim at the containment of the outbreak. Our previous study showed that if an Omicron epidemic is left untreated (and no spontaneous behaviour change was adopted by the population in response to the epidemic), the number of estimated deaths in mainland China could reach approximately 1.5 million deaths (i.e. approximately 100 per 100,000 individuals - in line with what was observed in the epidemic in Hong Kong). [23] This estimation further supports the key role that NPIs have played in reducing COVID-19 burden and mortality.

We found a high proportion of asymptomatic infections in Shanghai (90.7%, 95% CI: 90. 7-90.8%), which was greater than that estimated for the ancestral lineage (∼69%)[24] but consistent with other estimates for other SARS-CoV-2 variants in the presence of vaccination (∼85%). [25] Lower proportions of asymptomatic infections were reported in previous studies,[26] but those estimates were obtained in the absence of repeated screenings of the population. Moreover, it is important to note that the criteria adopted for the definition of asymptomatic infection may vary across locations and study periods. We estimated the overall IFR of the Omicron BA.2 outbreak in Shanghai to be 0.09% (95% CI: 0.09-0.10%), which is less than that of Hong Kong (a modelling study estimated an IFR of 0.19%). This difference may be explained by the shortage of hospital beds experienced by Hong Kong during the Omicron wave and differences in vaccination coverage. Vaccination coverage for the primary and booster doses is higher in Shanghai than in Hong Kong, especially among people aged 65 years or older (coverage of booster: 38% in Shanghai vs. 18% in Hong Kong before the Omicron outbreak). Among deceased cases reported in Hong Kong, greater than 70% of cases were never vaccinated. [3] Although the vaccines used in these two cities are not the same (inactivated vaccine in Shanghai, both inactivated and mRNA vaccines in Hong Kong), several studies have shown that the effectiveness against severe/critical COVID-19 disease or death is not significantly different between these two different platforms. More broadly, it is important to stress that increasing vaccine coverage could effectively reduce the clinical severity of COVID-19. We comprehensively summarized estimates of IFR as well as local vaccine-coverage data for several different settings, and we found that the IFR was lower in the setting with higher coverage of primary or booster vaccination, regardless of the type of circulating SARS-CoV-2 **(Appendix p2, p8-10)**. However, this analysis did not control for the different levels of natural infection and surveillance capacity in the study locations; this would require the collection of additional data, which are not readily available to us. Increasing vaccination coverage in Shanghai, especially for older people with a history of underlying chronic diseases, and overcoming vaccine hesitancy are critically important in preparation for possible future waves of COVID-19.

Our study has several limitations. First, due to the lack of reliable vaccination data in the general population, we could not estimate COVID-19 morbidity and mortality burden by vaccination status. Among SARS-CoV-2 infections with prior vaccination history, we could not retrieve data with further stratification by the timing and number of vaccine dose received, thus cannot assess dose-specific effects. Second, some asymptomatic infections who developed very mild symptom were not sent to the designated isolation hospital and were not counted as symptomatic cases, resulting in possible misclassification bias. Third, the analyzed data does not report information of possible underlying conditions of COVID-19 patients. Future studies leveraging individual-level data on underlying medical conditions would be needed to estimate conditional mortality risks as well as to control for biases in age-specific fatality risks emerging from the age-dependent prevalence of underlying medical conditions that could mediate the severity outcome of SARS-CoV-2 infections.

In conclusion, we estimated the disease burden and clinical severity of the Omicron BA.2 outbreak in Shanghai in February-June 2022. We found a lower burden and mortality of the outbreak compared to other settings and countries, showing the impact of successful outbreak containment in Shanghai. The estimated low clinical severity of this Omicron BA.2 epidemic in Shanghai highlighted the key contribution of vaccination and availability of hospital beds to reduce the risk of death.

## Data sharing

The data and code that support the findings of this study will be made available in GitHub upon manuscript acceptance.
